# The Suppression of Pin1-Alleviated Oxidative Stress through the p38 MAPK Pathway in Ischemia- and Reperfusion-Induced Acute Kidney Injury

**DOI:** 10.1155/2021/1313847

**Published:** 2021-07-30

**Authors:** Xiaojie Zhao, Dan Wang, Shanshan Wan, Xiuheng Liu, Wei Wang, Lei Wang

**Affiliations:** ^1^Department of Urology, Renmin Hospital of Wuhan University, Wuhan, 430060 Hubei, China; ^2^Department of Pharmacy Intravenous Admixture Service, Renmin Hospital of Wuhan University, Wuhan, 430060 Hubei, China; ^3^Department of Ophthalmology, Renmin Hospital of Wuhan University, Wuhan, 430060 Hubei, China; ^4^Department of Urology and Institute of Urology, The First Affiliated Hospital of Anhui Medical University, Anhui Province Key Laboratory of Genitourinary Diseases, Anhui Medical University, Hefei, 230022 Anhui, China

## Abstract

**Background:**

Pin1, as the peptidyl-prolyl isomerase, plays a vital role in cellular processes. However, whether it has a regulatory effect on renal ischemia and reperfusion (I/R) injury still remains unknown.

**Methods:**

The hypoxia/reoxygenation (H/R) model in human kidney (HK-2) cells and the I/R model in rats were assessed to investigate the role of Pin1 on I/R-induced acute kidney injury. Male Sprague-Dawley rats were used to establish the I/R model for 15, 30, and 45 min ischemia and then 24 h reperfusion, with or without the Pin1 inhibitor, to demonstrate the role of Pin1 in acute kidney injury. HK-2 cells were cultured and experienced the H/R model to identify the molecular mechanisms involved.

**Results:**

In this study, we found that Pin1 and oxidative stress were obviously increased after renal I/R. Inhibition of Pin1 with juglone decreased renal structural and functional injuries, as well as oxidative stress. Besides, Pin1 inhibition with the inhibitor, juglone, or the small interfering RNA showed significant reduction on oxidative stress markers caused by the H/R process in vitro. Furthermore, the results indicated that the expression of p38 MAPK was increased during H/R in vitro and Pin1 inhibition could reduce the increased expression of p38 MAPK.

**Conclusion:**

Our results illustrated that Pin1 aggravated renal I/R injury via elevating oxidative stress through activation of the p38 MAPK pathway. These findings indicated that Pin1 might become the potential treatment for renal I/R injury.

## 1. Introduction

Acute kidney injury (AKI), which is characterized by fast deterioration of renal function, becomes a worldwide public issue with high incidence and mortality [1]. Renal ischemia/reperfusion (I/R) injury, associated with partial nephrectomy, transplantation, shock, and cardiac bypass surgery, is a major risk for AKI and subsequent chronic kidney disease [2]. The mechanism of renal I/R injury is complex and involves a series of cellular processes, including oxidative stress, inflammation, apoptosis, and endoplasmic reticulum stress, which together lead to serious renal damage [3]. Previous research indicated that oxidative stress, characterized by rapid overproduction of reactive oxygen species (ROS), was considered one of the primary pathogeneses of I/R [4]. Therefore, it is urgent and beneficial for developing therapeutic strategy on reducing oxidative stress during renal I/R and thus attenuating AKI.

The phosphorylation of Ser/Thr residues on protein is a key mechanism to change their functional activity during the cellular process [5]. Pin1, as the peptidyl-prolyl isomerase, is an important regulator of phosphorylation-induced protein activation [6, 7]. Pin1 has important effect on the regulation of organic I/R injury. A previous report found that at early reperfusion times, Pin1 protein levels exhibited a rapid decline after cardiac ischemia, which was with a pattern resembling that of AKT protein, indicating a relationship between Pin1 loss and AKT stability decreased [8]. Another study showed that Pin1, which regulated p53 transactivation under stress, aggravated the pathogenesis via Notch signaling during ischemic stroke [9]. However, the effect of Pin1 on AKI induced by I/R injury remains unclear.

The mitogen-activated protein kinase (MAPK) pathway participates in numerous biological processes, including cell differentiation, cell proliferation, and adaptation to environmental stress [10]. p38 MAPK is a subclass of MAPKs, which is involved in the pathogenesis and progression of AKI [11]. Previous research indicated that the JNK/p38 MAPK pathway was activated following renal I/R, which was accompanied by the overproduction of ROS [12]. However, whether pin1 regulated p38 MAPK expression during AKI induced by I/R was still unclear. In the present study, we investigated whether Pin1 played an important role on renal I/R injury. Also, we tested the possible mechanisms that was involved in Pin1 regulating on oxidative stress.

## 2. Material and Methods

### 2.1. Animal

Male adult Sprague-Dawley (SD) rats (200–250 g) were provided by the center of experimental animals in the Medical College of Wuhan University. The animals were placed in a room with suitable temperature and humidity and free access to rat edibles and tap water. This experiment was authorized by the Ethics Committee of Renmin Hospital of Wuhan University, and the procedures were carried out in accordance with the principles of animal care of our university.

### 2.2. I/R Model Establishment

Rats were acclimated for a week and the model of renal I/R injury in rats was performed as previously reported [13]. After the rats were fully anesthetized with pentobarbital sodium (50 mg/kg, i.p.), they were subjected to the midline laparotomy. And then, all the rats experienced the right nephrectomy, followed by the left kidney vessels being clamped for 15, 30, and 45 min ischemia and then unclamped for 24 h reperfusion.

### 2.3. Animal Treatment

Rats were treated with various doses of the Pin1 inhibitor, juglone (2 or 10 mg/kg, once a day), three consecutive days before renal I/R injury establishment. The dimethyl sulfoxide (DMSO) group was injected with the equal DMSO solution as a control.

### 2.4. Serum Assays

Blood samples were collected to detect the level of blood urea nitrogen (BUN) and serum creatinine (Cr) through commercial kit instructions (Nanjing Jiancheng, China). The results were calculated and indicated through spectrophotometric methods.

### 2.5. Cell Treatment

The human kidney (HK-2) cells were cultured in DMEM (Invitrogen, USA) supplemented with 10% fetal bovine serum under 5% CO_2_ and 21% O_2_ at 37°C. To establish the cell model, the H/R process was performed as previously described [14]. Briefly, the HK-2 cells were incubated in the hypoxic condition (1% O_2_, 94% N_2_, and 5% CO_2_) for 3, 6, and 12 hours with the nonnutrient medium and then changed with normal medium and cultured in the normoxic condition for 6 hours. The control group was incubated in normal medium under normoxic condition.

### 2.6. Small Interfering RNA (siRNA) Transfection

For transfection of si-Pin1, the cells were transfected with two different si-RNA against Pin1 or si-NC for 48 h through lipofectamine 3000. Western blot was used to assess the effect of si-RNA against Pin1.

### 2.7. Histological Examinations

After the rats were sacrificed, renal tissue samples were fixed in 4% paraformaldehyde, embedded in paraffin, and incised with an average thickness of 4 *μ*m. Then, the samples were experiencing being deparaffinized, hydrated, and stained with hematoxylin and eosin (H&E). An established grading scale of 0–4, outlined by Jablonski et al. [15], was used for the histopathological assessment of I/R-induced damage.

### 2.8. RT-PCR

RNA was extracted with the RNAiso Plus (TaKaRa Biotechnology). Reverse transcriptase reactions were performed using a SuperScript First-strand Synthesis System (Invitrogen). Real-time PCR reactions were performed with *β*-actin as internal control. Gene levels were shown as fold change relative to control. The primers were included as follows:

R-Pin1: 5′-GCTCAGGCCGTGTCTACTACTTC-3′ (F); 5′-TCCGAGATTGGCTGTGCTTC-3′ (R); R-*β*-actin: 5′-TGCTATGTTGCCCTAGACTTCG-3′ (F); 5′-GTTGGCATAGAGGTCTTTACGG-3′ (R); H-Pin1: 5′-TCAACCACATCACTAACGCCAG-3′ (F); 5′-GCAAACGAGGCGTCTTCAAAT-3′ (R); H-*β*-actin: 5′-CACCCAGCACAATGAAGATCAAGAT-3′ (F); and 5′-CCAGTTTTTAAATCCTGAGTCAAGC-3′ (R).

### 2.9. Western Blotting

Kidney tissue was collected and then protein was extracted and quantified. Briefly, the sample was separated and then transferred to the polyvinylidene difluoride membrane. Subsequently, it was blocked with 5% nonfat milk and then incubated at 4°C overnight with primary antibodies against Pin1 (#192036, Abcam), 4-hydroxynonenal (4-HNE) (#46545, Abcam), COX2 (cyclooxygenase 2) (#179800, Abcam), myeloperoxidase (MPO) (#208670, Abcam), p38 (#31828, Abcam), p-p38(#4822, Abcam), and *β*-actin (#BA2305, Boster Biological Technology). Then, it was washed and incubated with secondary antibody. Specific bands were detected and the densities were quantified using ImageJ software.

### 2.10. Measurement of Malondialdehyde (MDA) and Superoxide Dismutase (SOD)

The detection of SOD activity and MDA content were performed through the instructions of the Nanjing Jiancheng Bioengineering Institute. Cell lysates from in vivo and in vitro experiments were collected and detected the level of SOD and MDA. The specific procedures were according with the manufacturer's direction.

### 2.11. ROS Production Detection

The ROS levels were determined based on our previous study [16] through the commercial kit instruction (Nanjing Jiancheng, China). The samples were incubated with dichloro-dihydro-fluorescein diacetate (20 *μ*M) at room temperature for 30 min, and the result of the ROS level was quantified through flow cytometry.

### 2.12. Detection for the Production of Hydrogen Peroxide (H_2_O_2_)

The renal tissue and HK-2 cells were perfused and homogenized. And then, hydrogen peroxide was detected through the commercial kit instruction (Nanjing Jiancheng, China). The results were shown as fold change relative to the control group.

### 2.13. DHE Staining

Frozen kidney sections (4 *μ*M) were stained with dihydroethidium (DHE) (2 *μ*mol/L, Sigma) in a light-protected humidified chamber at 37°C for 15 min. The images were visualized using a fluorescence microscope (Olympus IX51).

### 2.14. Statistical Analysis

All data was expressed as mean ± standard error of the mean (SEM). Statistical analyses included two-way analysis of variance and the Student–Newman–Keuls test. Statistically significant differences were considered when *p* < 0.05.

## 3. Results

### 3.1. Pin1 Expression Was Elevated during Renal I/R

**Figure 1:**
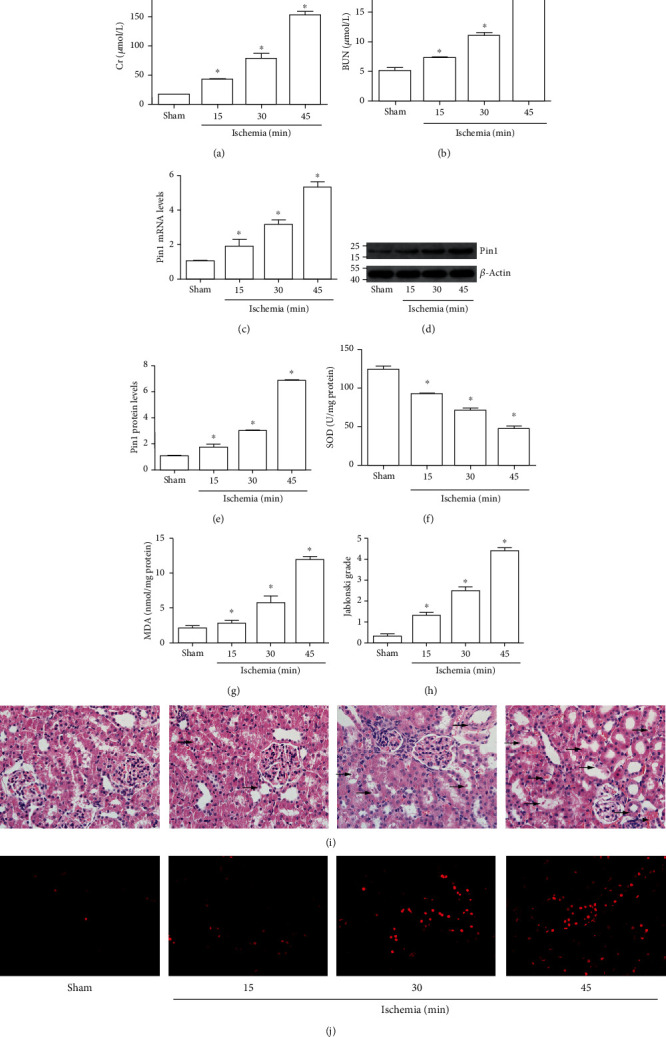
Pin1 expression was increased during renal I/R injury in rats. The model was established with 15, 30, and 45 min ischemia and 24 h reperfusion. (a, b) The Cr and BUN levels in serum were detected. (c) The Pin1 mRNA level was increased in RT-PCR. (d, e) The Pin1 protein level was increased in Western blot, and the quantification was performed. (f, g) The SOD activity and MDA content were detected after I/R injury in rats. (h, i) The pathological change was examined by H&E staining and its quantification (×400). (j) The renal ROS level was examined by DHE staining (×400) (*n* = 5). The values were presented as mean ± SEM. ^∗^*P* < 0.05 vs. the sham group.

The levels of Bun and Cr were continuing to increase as the ischemic time extended (Figures 1(a) and 1(b)). Also, the levels of Pin1 were examined by RT-PCR and Western blot and the results showed that Pin1 mRNA and the protein level were continuing to increase as the extension of ischemic time, especially at 45 min ischemia (Figures 1(c)–1(e)). Then, we found that oxidative stress was increased during renal I/R. The results showed that SOD activity (Figure 1(f)) continued to decrease and MDA content (Figure 1(g)) continued to increase in the course of I/R progress, especially at 45 min ischemia. H&E staining (Figures 1(h) and 1(i)) and DHE staining (Figure 1(j)) indicated that renal I/R injury exhibited acute tubular damage and increased ROS level at ischemia 45 min. Therefore, we chose ischemia 45 min and reperfusion 24 h in the following experiments.

### 3.2. Pin1 Inhibition Protected against Renal Injury Induced by I/R

**Figure 2:**
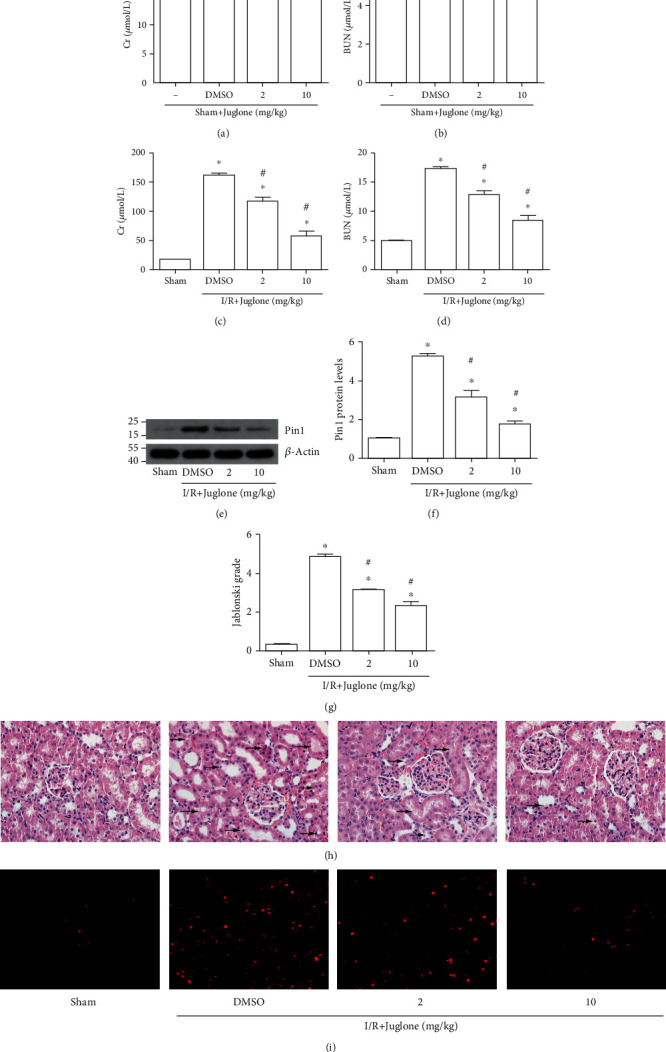
The Pin1 inhibitor, juglone, prevented renal tissue against I/R injury in rats. The model was established with 45 min ischemia and then 24 h reperfusion. (a, b) The effect of juglone at different concentrations (2 mg/kg, 10 mg/kg) on the Cr and BUN levels in the sham group. (c, d) The effect of juglone at different concentrations (2 mg/kg, 10 mg/kg) on the Cr and BUN levels in the I/R group. (e, f) The effect of juglone at different concentrations (2 mg/kg, 10 mg/kg) on Pin1 expression in rats and the quantification. (g, h) The effect of juglone at different concentrations (2 mg/kg, 10 mg/kg) on the structure change was examined by H&E staining and its quantification (×400). (i) The effect of juglone on oxidative stress was examined by DHE staining (×400) (*n* = 5). The values were presented as mean ± SEM. ^∗^*P* < 0.05 vs. the sham group and ^#^*P* < 0.05 vs. the I/R group.

Firstly, different concentrations of the Pin1 inhibitor, juglone, were applied in the sham group. The results indicated that juglone did not affect renal function, as BUN and Cr levels did not have difference between groups (Figures 2(a) and 2(b)). Then, we found that the increased BUN and Cr levels induced by I/R obviously decreased after juglone treatment (Figures 2(c) and 2(d)). The results of Western blot showed that the treatment of juglone could inhibit the elevated expression of Pin1 caused by renal I/R (Figures 2(e) and 2(f)). Besides, H&E staining showed that renal I/R injury exhibited acute tubular damage, including the loss of the brush border and tubular dilatation in the proximal tubules (Figures 2(g) and 2(h)). However, the different concentration of juglone protected renal tissue from acute tubular damage, with better effects at 10 mg/kg. The DHE staining also showed that juglone could reduce the elevated ROS levels induced by I/R (Figure 2(i)).

### 3.3. Pin1 Inhibition Attenuated Oxidative Stress Caused by Renal I/R

**Figure 3:**
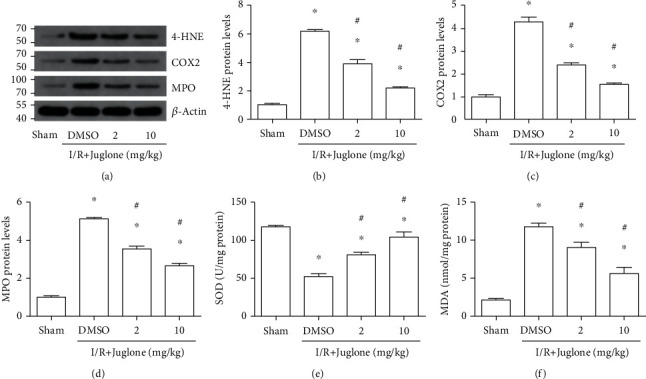
The Pin1 inhibitor, Juglone, reduced oxidative stress induced by renal I/R in rats. The model was established with 45 min ischemia and then 24 h reperfusion. (a–d) The regulatory effect of the Pin1 inhibitor on 4-HNE, COX2, and MPO expression after renal I/R, and quantification was performed. (e) The effect of the Pin1 inhibitor on the SOD activity after renal I/R. (f) The effect of the Pin1 inhibitor on MDA content after renal I/R (*n* = 5). The values were presented as mean ± SEM. ^∗^*P* < 0.05 vs. the sham group and ^#^*P* < 0.05 vs. the I/R group.

Next, we investigated the relationship of Pin1 and oxidative stress induced by renal I/R injury. Western blot results showed that the 4-HNE, COX2, and MPO expression was elevated after renal I/R injury and Pin1 inhibition could alleviate their expression (Figures 3(a)–3(d)). Also, the results indicated that the reduced SOD level (Figure 3(e)) and the elevated MDA content (Figure 3(f)) induced by renal I/R were reversed by the Pin1 inhibitor. Therefore, it indicated that Pin1 inhibition might alleviate oxidative stress induced by renal I/R.

### 3.4. Pin1 Level and Oxidative Stress Were Elevated during H/R In Vitro

**Figure 4:**
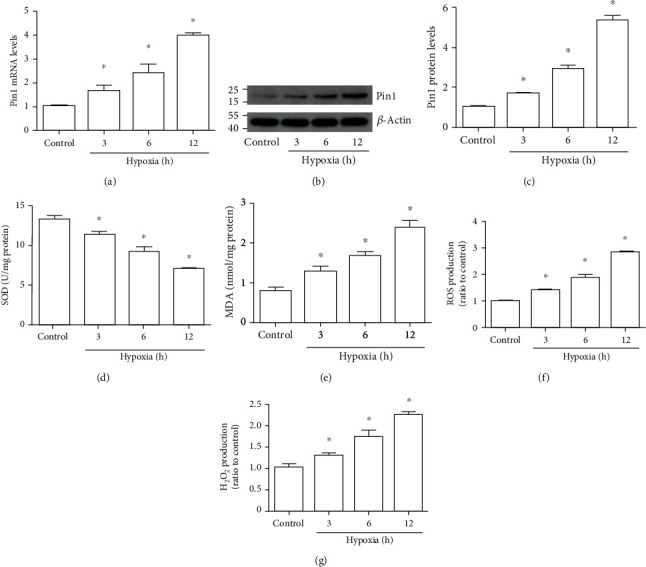
Pin1 expression and oxidative stress were elevated during the H/R process in vitro. The model was established with 3, 6, and 12 h hypoxia and 6 h reoxygenation. (a) The mRNA was detected by RT-PCR after the H/R process. (b, c) Western blot was used to detect Pin1 expression and the quantification was analyzed. (d–g) The SOD, MDA, ROS, and H_2_O_2_ levels were detected after H/R injury in vitro (*n* = 5). The values were presented as mean ± SEM. ^∗^*P* < 0.05 vs. the control group.

RT-PCR and Western blot results indicated that the Pin1 mRNA and protein levels were increased after different hypoxia time and reoxygenation 6 h in HK-2 cells, especially at hypoxia 12 h (Figures 4(a)–4(c)). We also found that the SOD activity (Figure 4(d)) continued to decrease during the H/R course in HK-2 cells. However, the MDA content (Figure 4(e)), ROS (Figure 4(f)), and H_2_O_2_ production (Figure 4(g)) were increased in response to H/R. So, we chose 12 h hypoxia and 6 h reoxygenation in the following experiments.

### 3.5. Juglone Decreased Oxidative Stress Caused by H/R In Vitro

**Figure 5:**
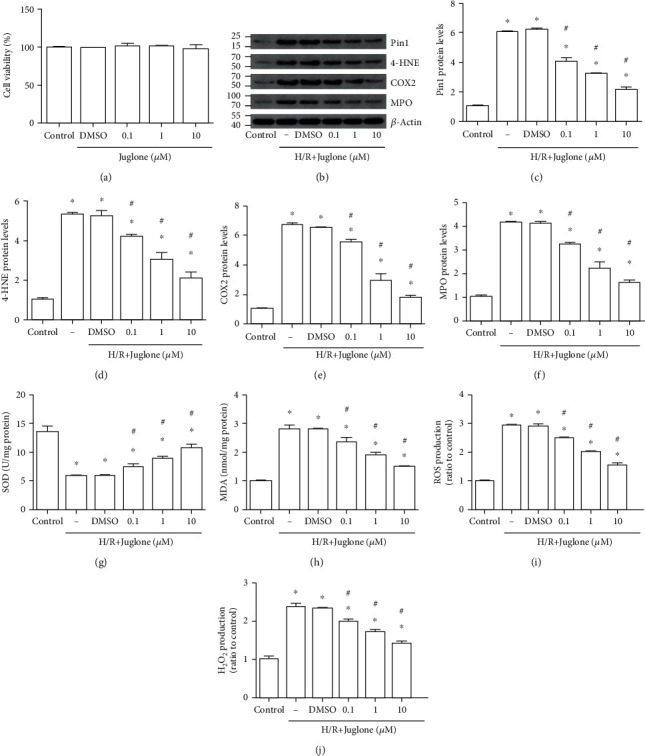
The Pin1 inhibitor, Juglone, regulated oxidative stress caused by H/R injury in vitro. The model was established with 12 h hypoxia and 6 h reoxygenation. HK-2 cells were treated with the Pin1 inhibitor (0.1,1, and 10 *μ*M) for 1 h and then experienced the H/R process. (a) The effect of different concentrations of juglone on the control group. (b–f) Western blot was used to detect the expression of Pin1, 4-HNE, COX2, and MPO and quantification was performed. (g–j) The SOD, MDA, ROS, and H_2_O_2_ levels were detected after the H/R process in vitro (*n* = 5). The values were presented as mean ± SEM. ^∗^*P* < 0.05 vs. the control group and ^#^*P* < 0.05 vs. the H/R group.

Firstly, the different concentration of juglone was used in the control group and the CCK-8 results showed that cell viability had no differences between groups (Figure 5(a)). Western blot results showed that Pin1, 4-HNE, COX2, and MPO expression was increased after H/R and the different concentration of juglone could decrease their expression in HK-2 cells (Figures 5(b)–5(f)), especially at 10 *μ*M. Next, the results indicated that the reduced SOD level (Figure 5(g)) and the elevated MDA content (Figure 5(h)), ROS (Figure 5(i)), and H_2_O_2_ production (Figure 5(j)) induced by H/R were reversed by inhibition of Pin1 in HK-2 cells. Therefore, we chose juglone 10 *μ*M in the following experiments.

### 3.6. Pin1 Inhibition Reduced Oxidative Stress Caused by H/R In Vitro

**Figure 6:**
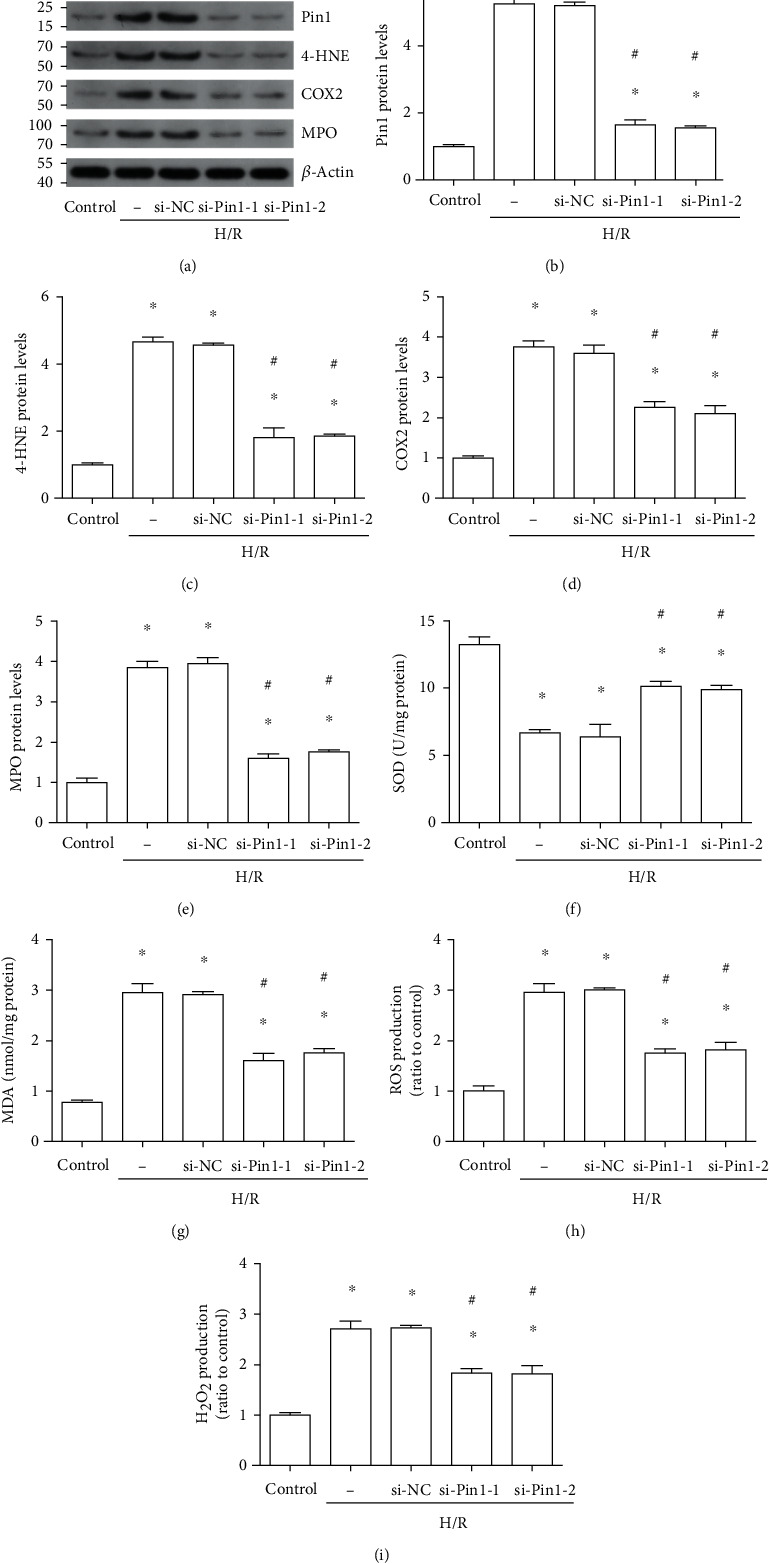
Pin1 silence decreased oxidative stress induced by H/R in vitro. The H/R model was established with 12 h hypoxia and 6 h reoxygenation. The cells were transfected with si-NC or two different si-Pin1 for 24 h and then experienced H/R. (a–e) Western blot was used to detect the expression of Pin1, 4-HNE, COX2, and MPO and quantification was performed. (f–i) The SOD, MDA, ROS, and H_2_O_2_ levels were detected after H/R in vitro (*n* = 5). The values were presented as mean ± SEM. ^∗^*P* < 0.05 vs. the control group and ^#^*P* < 0.05 vs. the si-NC group.

Two different siRNAs against Pin1 were performed to demonstrate the role of Pin1 in oxidative stress induced by H/R in vitro. The results of Western blot showed that si-RNA against Pin1 could obviously alleviate the expression of Pin1, 4-HNE, COX2, and MPO, which was elevated after H/R in HK-2 cells (Figures 6(a)–6(e)). Also, the results indicated that compared with the si-NC group, the SOD level (Figure 6(f)) was obviously increased and the MDA content (Figure 6(g)), ROS (Figure 6(h)), and H_2_O_2_ production (Figure 6(i)) were decreased by si-RNA against Pin1 in HK-2 cells.

### 3.7. p38 MAPK Involved in the Regulation of Pin1 on Oxidative Stress Caused by H/R In Vitro

**Figure 7:**
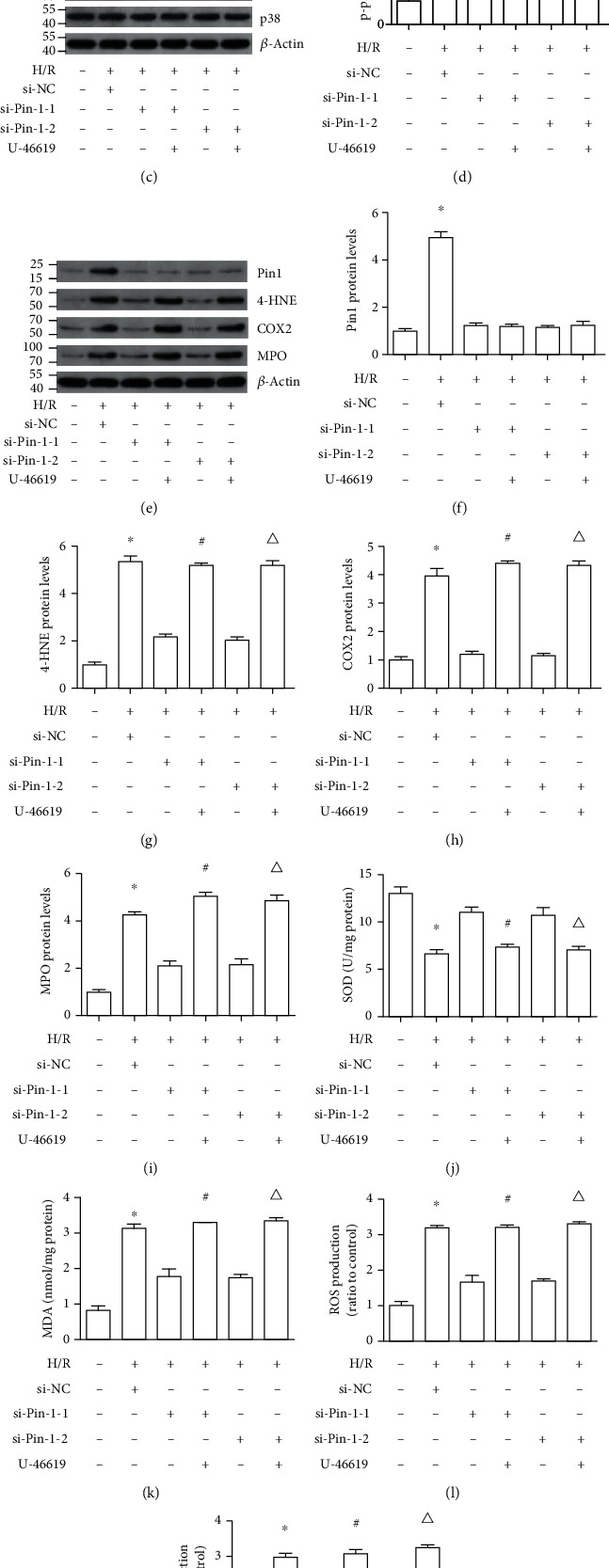
Pin1 aggravates oxidative stress caused by H/R injury via activation of p38 MAPK. The H/R model was established with 12 h hypoxia and 6 h reoxygenation. The cells were transfected with si-NC or two different si-Pin1 for 24 h and then experienced the H/R process, with or without treatment with the p38 MAPK activator (5 *μ*M). (a, b) Western blot was used to detect the expression of p-p38/p38 after Pin1 silence and quantification was performed. (c, d) The expression of p-p38/p38 after Pin1 silence and its quantification, with or without treatment with the p38 MAPK activator. (e–i) Western blot was used to detect the expression of Pin1, 4-HNE, COX2, and MPO and quantification was performed. (j–m) The SOD, MDA, ROS, and H_2_O_2_ levels were detected (*n* = 5). The values were presented as mean ± SEM. ^∗^*P* < 0.05 vs. the control group; ^#^*P* < 0.05 vs. the si-Pin1-1 group; ^△^*P* < 0.05 vs. the si-Pin1-2 group.

It was reported that p38 MAPK was activated in renal I/R injury. In the present study, we found that phosphorylated p38 (p-p38) was largely inhibited by si-Pin1 compared with si-NC (Figures 7(a) and 7(b)). Next, we applied the p38 MAPK activator, U-46619, to investigate the association between Pin1 and p38 MAPK. Western blot results indicated that the inhibited p-p38 by two different si-Pin1 could be reversed by U-46619 (Figures 7(c) and 7(d)). Next, we found that the expression of Pin1 did not have a difference after treatment with U-46619; however, the inhibited expression of 4-HNE, COX2, and MPO by si-Pin1 was reversed by U-46619 treatment (Figures 7(e)–7(i)). Also, compared with two different si-Pin1 groups, the changed levels of the SOD activity (Figure 7(j)), MDA content (Figure 7(k)), ROS (Figure 7(l)), and H_2_O_2_ production (Figure 7(m)) were reversed after U-46619 treatment. These results suggested that Pin1 regulated oxidative stress during H/R in HK-2 cells that depended on the p38 MAPK pathway.

### 3.8. Inhibition of Pin1 Attenuated p38 MAPK Activation Induced by Renal I/R

**Figure 8:**
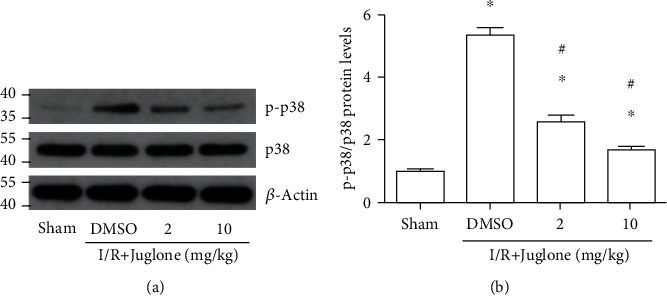
The Pin1 inhibitor, Juglone, ameliorates p38 MAPK activation induced by renal I/R in rats. The model was performed with 45 min ischemia and 24 h reperfusion. (a–b) The regulatory effect of the Pin1 inhibitor on p-p38/p38 expression after renal I/R in rats and quantification performed (*n* = 5). The values were presented as mean ± SEM. ^∗^*P* < 0.05 vs. the sham group and ^#^*P* < 0.05 vs. the I/R group.

Western blot results showed that p-p38 MAPK was increased after renal I/R in rats; however, the expression was inhibited by treatment with juglone, a Pin1 inhibitor (Figures 8(a) and 8(b)).

## 4. Discussion

In this study, we investigated the role of Pin1 in renal I/R injury and the possible mechanism. The results showed that Pin1 had important effects on the regulation of renal I/R in vivo. Firstly, we found that the inhibition of Pin1 could alleviate renal injury induced by I/R *in vivo* and *in vitro*. Besides, oxidative stress induced by H/R depended on Pin1 levels in HK-2 cells and inhibition of Pin1 using siRNA or the specific inhibitor blocked oxidative stress caused by H/R in vitro. Furthermore, we also found that ROS generation was modulated by Pin1 through p38 MAPK activation. So, our study demonstrated that Pin1 might become a target for treatment of renal I/R injury.

I/R is the common pathophysiological process that leads to renal tubular epithelial cell death and subsequently rapid deterioration of renal function [17]. Experimental renal I/R models are critical for the researchers to investigate the pathogenesis and explore the development of effective therapeutics. The in vivo models of renal I/R included both renal pedicle clamping (bilateral I/R) and one renal pedicle clamping (unilateral I/R) or unilateral I/R with contralateral nephrectomy [18]. Also, the ischemic time and reperfusion period were different which depended on the species or experimental purpose. In our research, the renal I/R model on rats was established, with ischemic time ranging from 15 min to 45 min and 24 h reperfusion. The results showed that oxidative stress became more serious as the ischemic time prolonged, especially at 45 min ischemia, which was consistent with a previous study, demonstrating that oxidative stress might be important in renal I/R injury.

Pin1, a key regulatory mediator, is involved in various cellular processes through specifically recognizing pSer/pThr-Pro motifs and inducing conformational changes to control protein function [19]. Previous studies focused more on the effect of Pin1 on tumorigenesis and progression; however, it also played an important role in organic ischemic injury, including cardiac, hepatic, cerebral, and intestinal I/R [8, 19–21]. In the present study, it indicated that Pin1 expression continued to increase as the ischemic time extended, especially at 45 min ischemia. The H/R process was performed to mimic the in vitro model and the results indicated that Pin1 expression was elevated as hypoxia time prolonged, especially at hypoxia 12 h, which was consistent with our in vivo results. The inhibition of Pin1 could protect renal tissue and function against I/R injury.

ROS, at low concentrations, serves to regulate various cellular signaling pathways and modulate cell fate and proliferation, thus maintaining cellular and tissue homeostases. However, the high ROS level is also detrimental to physiological function. Oxidative stress results from the accumulation of high levels of ROS and plays the vital role during the organic I/R injury [22]. A previous study indicated that trehalose could protect the kidney against I/R injury through blocking oxidative stress [23]. In our research, the results indicated that inhibition of Pin1 could reverse the elevated 4-HNE, COX2, and MPO expression induced by renal I/R, as well as the reduced SOD activity and elevated MDA content. To demonstrated the results in vivo, we also used si-RNA against Pin1 and juglone, a Pin1-specific inhibitor, through the H/R model in vitro. The results in vitro were consistent with those in vivo that inhibition of Pin1 could alleviate the expression of 4-HNE, COX2, and MPO; MDA content; ROS; and H_2_O_2_ production and increased SOD activity after H/R in HK-2 cells. Our results indicated that Pin1 inhibition could alleviate renal I/R injury through the regulation of oxidative stress.

P38-MAP kinase, a critical component of MAPK systems, is involved in the modulation of cellular function [24]. Different stimuli could activate p38-MAPK and aggravate I/R injury [25]. A recent study indicated that p38-MAPK caused kidney I/R injury through regulation of redox stress and cell apoptosis, which suggested that it might be a potential target of AKI [26]. The relationship of Pin1 and p-p38 had already been reported and the mechanism was complex. Coimmunoprecipitation analysis showed that Pin1 could bind to p-p38, which implied that the p-p38 MAPK might be a substrate of Pin1. Then, with GST pulldown experiment, it showed that Pin1 could not directly bind to p-p38 MAPK in vitro, suggesting that Pin1 might affect p38 MAPK through kinases or other proteins [27]. In this study, we found that the activation of p38 MAPK was induced by I/R injury and inhibition of Pin1 could suppress p38 MAPK activation in vivo and in vitro. Besides, with the p38 MAPK activator, we further demonstrated that the decreased p-p38 level was elevated in response to U-46619, compared with si-Pin1 only. In addition, the results also indicated that the changed expression of 4-HNE, COX2, and MPO induced by two different si-Pin1, as well as the changed levels of SOD activity, MDA content, ROS, and H_2_O_2_ production, could be reversed by U-46619, which demonstrate that Pin1 regulated oxidative stress through p38 MAPK. The mechanism of Pin1 regulating on the activation of p38 MAPK might be through modulation of protein kinases.

## 5. Conclusion

In summary, we found that Pin1 inhibition could protect the renal tissue against I/R injury and prevent kidney tissue through modulating p38 MAPK-mediated ROS production. Overall, the present study indicated that Pin1 might become a potential treatment for renal I/R injury.

## Data Availability

The datasets in this study are available from the corresponding author.

## Abbreviations

Pin1: Peptidyl-prolyl cis/trans isomerase
SD: Sprague-Dawley
I/R: Ischemia and reperfusion
HK-2: Human kidney
BUN: Blood urea nitrogen
H/R: Hypoxia and reoxygenation
AKI: Acute kidney injury
4-HNE: 4-Hydroxynonenal
COX2: Cyclooxygenase 2
MPO: Myeloperoxidase
Cr: Creatinine
siRNA: Small interfering RNA
DMSO: Dimethyl sulfoxide
ROS: Reactive oxygen species
NC: Negative control
H&E: Hematoxylin and eosin
H_2_O_2_: Hydrogen peroxide
MAPK: Mitogen-activated protein kinases
qRT-PCR: Real-time quantitative reverse transcription-polymerase chain reaction.
